# Postmenopausal Enlargement of a Presumed Leiomyoma Revealing STUMP: A Diagnostic Pitfall with Important Clinical Implications—A Case Report

**DOI:** 10.3390/diagnostics16071075

**Published:** 2026-04-02

**Authors:** Nenad Rakic, Stefan Ivanovic, Milica Ivanovic, Lidija Tulic, Milos Milincic, Tatjana Dosev, Nikola Jovic, Neda Arsenijevic, Jovana Joksimovic Jovic

**Affiliations:** 1 Senta General Hospital, 24400 Senta, Serbia; 2 Obstetrics and Gynecology Clinic “Narodni Front”, 11000 Belgrade, Serbia; 3 Clinic for Gynecology and Obstetrics, University Clinical Center of Serbia, 11000 Belgrade, Serbia; 4 Faculty of Medicine, University of Belgrade, 11000 Belgrade, Serbia; 5 Department of Gynecology and Obstetrics, University of Kragujevac, 34000 Kragujevac, Serbia; 6 Faculty of Medical Sciences, University of Kragujevac, 34000 Kragujevac, Serbia; 7 Department of Physiology, Faculty of Medical Sciences, University of Kragujevac, 34000 Kragujevac, Serbia

**Keywords:** uterine STUMP, postmenopausal bleeding, uterine leiomyoma, differential diagnosis, histopathology, gynecologic tumors

## Abstract

**Background and Clinical Significance:** Uterine smooth muscle tumors range from benign leiomyomas to highly aggressive leiomyosarcomas. Smooth muscle tumors of uncertain malignant potential (STUMP) represent an intermediate and diagnostically challenging category defined by borderline or discordant histological features. Their clinical management remains complex due to limited possibilities for reliable preoperative differentiation and the absence of clearly established surveillance protocols. The situation becomes particularly sensitive in postmenopausal patients, in whom tumor growth or abnormal bleeding raises concern for malignancy. **Case Presentation:** We report a 66-year-old postmenopausal woman presenting with persistent uterine bleeding and interval growth of a previously presumed leiomyoma. Transvaginal ultrasound demonstrated a heterogeneous intramural mass measuring approximately 5–7 cm, while endometrial sampling revealed inactive, atrophic endometrium without evidence of malignancy. Given the patient’s postmenopausal status and progressive symptoms, total abdominal hysterectomy with bilateral adnexectomy was performed. Histopathological examination identified moderate cytological atypia, focal coagulative tumor necrosis, and mitotic activity of up to five mitoses per ten high-power fields, findings insufficient for leiomyosarcoma but exceeding those expected for a benign leiomyoma. A diagnosis of STUMP was established. Postoperative staging showed no residual or metastatic disease, and structured long-term follow-up was initiated. **Discussion:** This case illustrates the limitations of current preoperative diagnostic tools in distinguishing between benign and borderline or malignant uterine smooth muscle tumors. Clinical presentation, imaging, and endometrial sampling were not predictive of the final diagnosis. In postmenopausal women, enlargement of a presumed leiomyoma should prompt careful evaluation, as histological assessment after complete surgical removal often remains the only reliable method of diagnosis. The unpredictable biological behavior of STUMP and reported cases of late recurrence support the need for prolonged surveillance, even after apparently adequate surgical treatment. **Conclusions:** STUMP remains primarily a postoperative diagnosis and represents a persistent gray zone in gynecologic oncology. Postmenopausal tumor growth and abnormal bleeding warrant an individualized and cautious approach. Careful histopathological evaluation and long-term follow-up are essential to ensure early detection of possible recurrence and optimal patient management.

## 1. Introduction

Uterine smooth muscle tumors are the most common mesenchymal neoplasms of the female genital tract and include a broad spectrum of lesions, ranging from benign leiomyomas (LMs) to highly malignant leiomyosarcomas (LMS) [1]. LMs are found in up to 60–70% of women of reproductive age and can be detected in more than 70–80% of uteri at histopathological examination, with a higher prevalence and earlier onset consistently reported among women of African-American descent [2]. LMs are among the most frequent benign tumors in women of reproductive and menopausal age and, in the majority of cases, usually demonstrate slow growth and a favorable clinical course. In contrast, LMS are rare but biologically aggressive neoplasms associated with early hematogenous dissemination and poor survival outcomes. LMS cases account for approximately 1–2% of all uterine malignancies. The estimated annual incidence of LMS is approximately 0.4–0.8 per 100,000 women, underscoring its rarity compared with benign LMs [1,3,4]. The differentiation between benign and malignant uterine smooth muscle tumors is based on a histological assessment of cytological atypia, mitotic activity, and the presence of coagulative tumor necrosis, which serve as the cornerstone of modern classification and diagnostic practice in gynecologic pathology [3]. Despite clearly defined histological parameters, certain tumors exhibit morphological features that preclude reliable classification as either benign or malignant. These lesions are categorized as smooth muscle tumors of uncertain malignant potential (STUMP). STUMP represents a very small fraction (<1%) of uterine smooth muscle tumors. Most reported cases occur in women between 40 and 55 years of age, although STUMP may also be diagnosed in postmenopausal patients. These tumors occupy a morphological and clinical “gray zone,” and their identification is based on a combination of histological parameters that exceed typical benign findings but do not reach the threshold for malignancy [2,3]. The clinical presentation is typically characterized by the presence of a tumor mass and/or vaginal bleeding. Abnormal uterine bleeding represents the leading presenting symptom and is described in 47.1% of cases [5]. The clinical significance of STUMP tumors arises from their unpredictable biological behavior and the absence of standardized guidelines for optimal treatment and follow-up. A definitive diagnosis is most often established only after surgical management and detailed histopathological evaluation, while reliable preoperative differentiation among LMs, STUMP tumors, and LMS remains limited. Histological criteria cannot fully predict the clinical course, and assessment of malignant potential, therefore, continues to represent a challenge in routine clinical practice [1,2]. The absence of a clear consensus on treatment strategy and follow-up further complicates clinical decision-making in these patients [6]. In this report, we present a case of STUMP in a postmenopausal patient that clinically manifested with persistent uterine bleeding and was initially interpreted as an LM. The case highlights the diagnostic dilemmas in preoperative assessment, the importance of detailed histopathological analysis, and the need for an individualized therapeutic approach and long-term follow-up in accordance with current evidence from the literature.

## 2. Case Presentation

**Figure 1:**
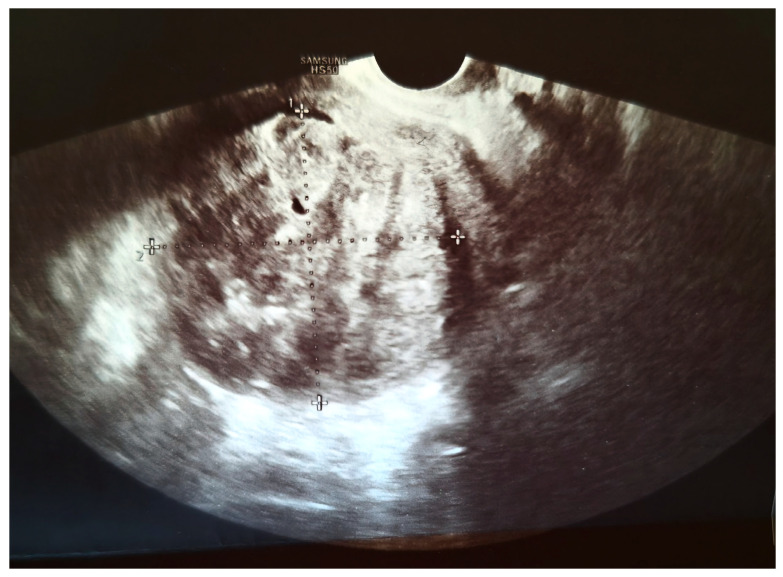
Transvaginal ultrasound image (transverse plane) showing a uterine mass measuring 64 × 62 mm.

**Figure 2:**
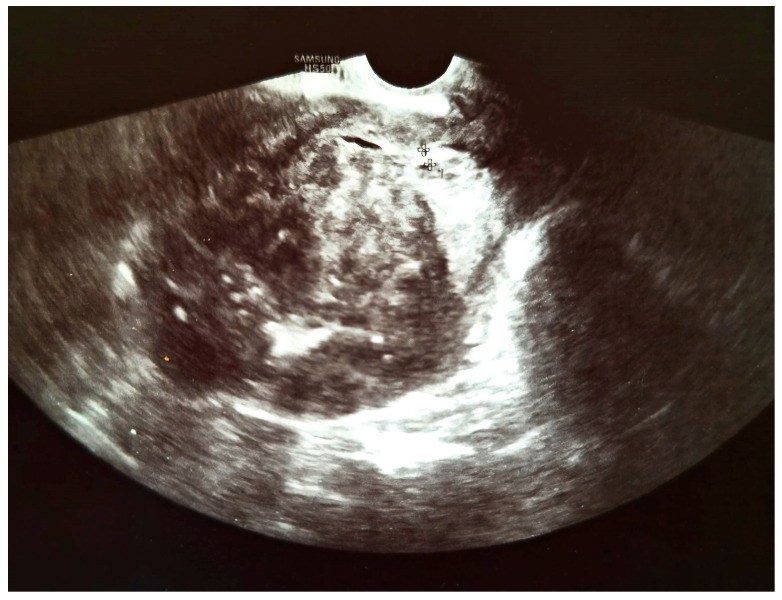
Transvaginal ultrasound image (longitudinal plane) demonstrating an endometrial thickness of 3.4 mm.

**Figure 3:**
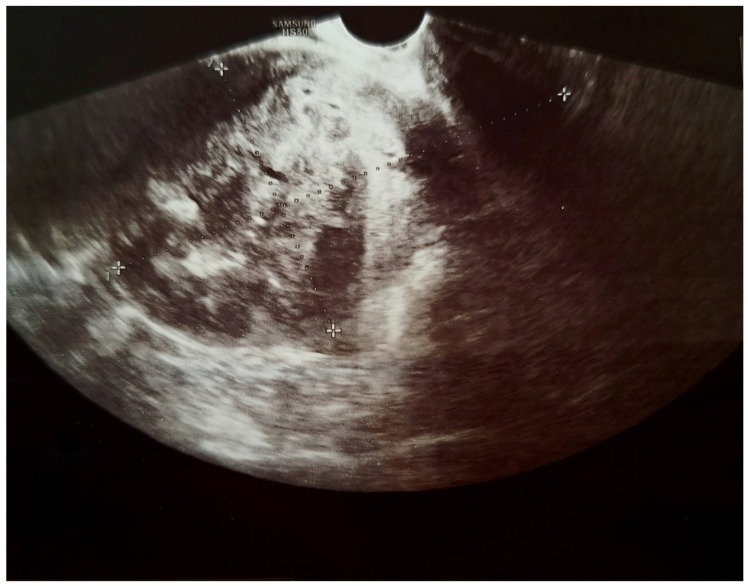
Transvaginal ultrasound image (longitudinal plane) showing an anteverted and anteflexed uterus measuring 106.3 × 65 mm.

**Figure 4:**
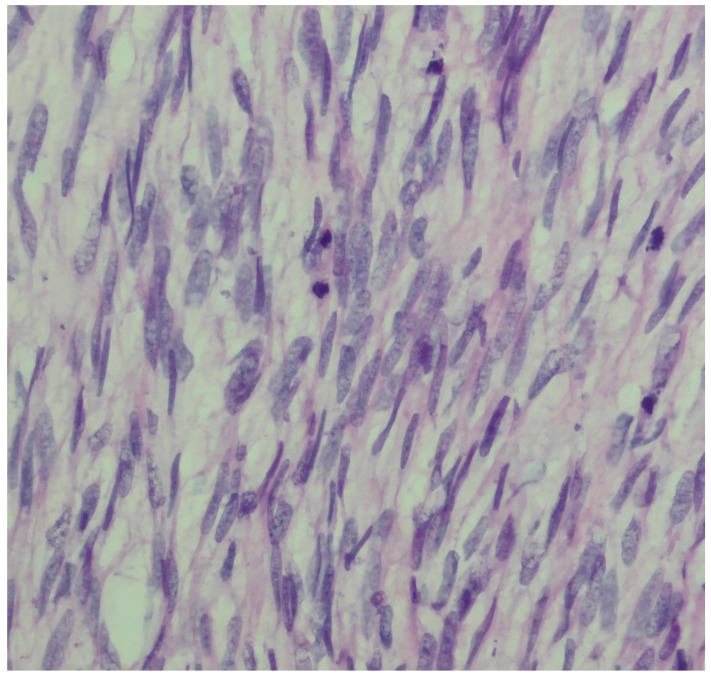
High-power view (×600, H&E) showing a highly cellular spindle cell tumor with mild-to-moderate cytological atypia and occasional mitotic figures.

**Figure 5:**
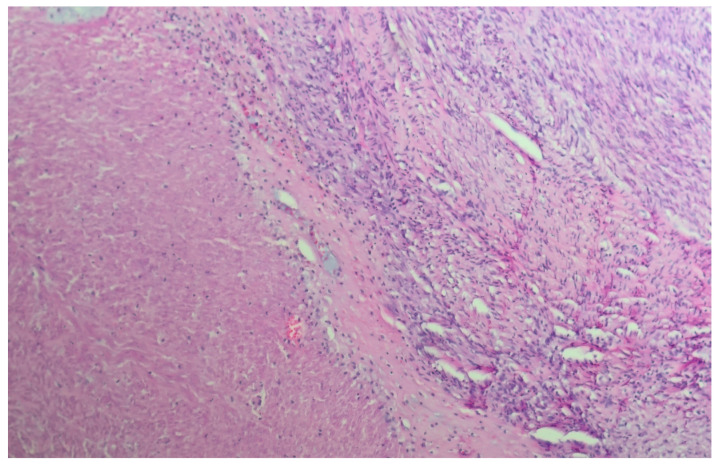
Low-power view (×100, H&E) demonstrating areas consistent with coagulative tumor cell necrosis, with an abrupt transition between viable and necrotic tumor tissue.

**Figure 6:**
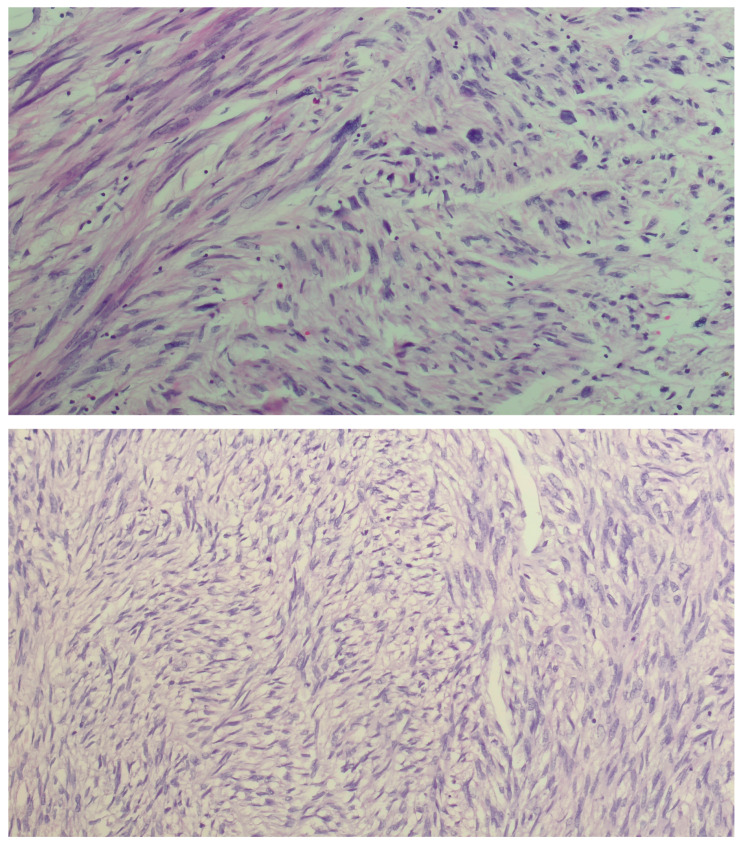

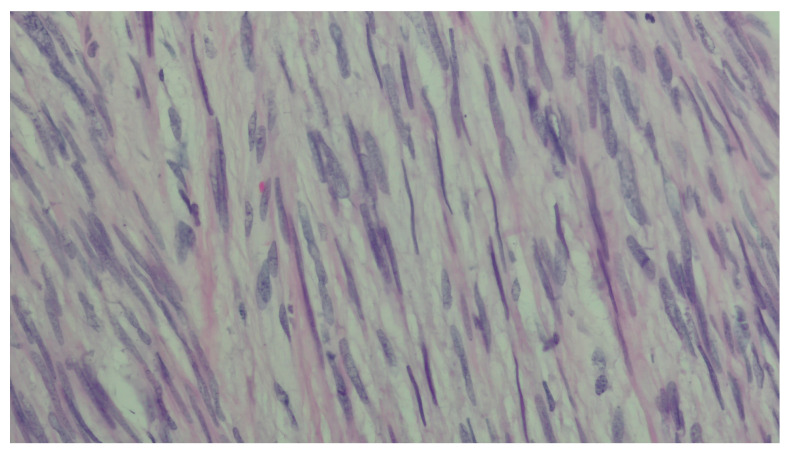
Intersecting fascicles of smooth muscle tumor cells with mild to moderate cytological atypia (×200–×400, H&E).

**Figure 7:**
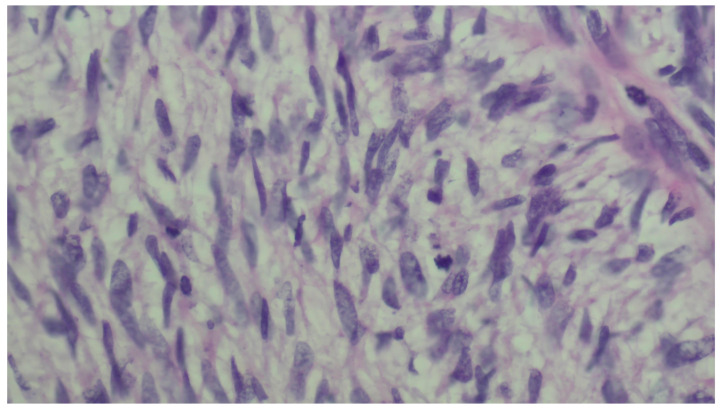
High-power view (×600, H&E) showing spindle-shaped smooth muscle tumor cells with moderate cytological atypia.

**Figure 8:**
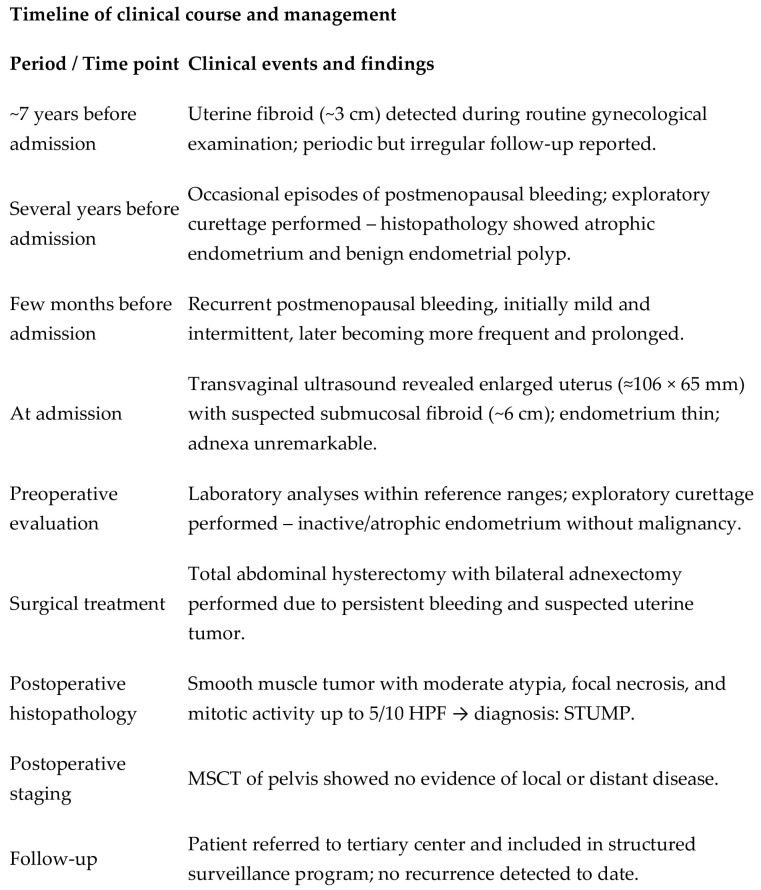
Schematic timeline summarizing the clinical course, diagnostic procedures, therapeutic interventions, and follow-up.

We present a 66-year-old woman. She has been postmenopausal for approximately ten years. She was referred to the Department of Gynecology for evaluation of persistent postmenopausal uterine bleeding. Her obstetric history included two vaginal deliveries and one spontaneous miscarriage. She had a history of well-controlled arterial hypertension managed with antihypertensive therapy. Family history was unremarkable. In the patient’s history, she had been aware of a uterine “fibroid” for approximately seven years. She reported that the lesion had initially measured around 3 cm in diameter. But no previous medical documentation or imaging studies were available for comparison. During this period, she remained asymptomatic, and no active treatment had been undertaken. Several years prior to the present admission, she experienced an earlier episode of postmenopausal bleeding. At that time, exploratory curettage was performed, and histopathological examination revealed an atrophic endometrium and an endometrial polyp. Following this intervention, the patient remained asymptomatic for a prolonged period. In the months preceding hospitalization, vaginal bleeding recurred. Initially mild and intermittent, it gradually became more frequent and prolonged, prompting further evaluation. Transvaginal ultrasound (TVUS) examination of the uterus in both longitudinal and transverse planes demonstrated an enlarged uterus measuring approximately 106 mm × 65 mm, with a heterogeneous intramural mass located on the posterior uterine wall (FIGO type 2–5), measuring approximately 6 cm × 6 cm and suggestive of an LM. The endometrium was thin and consistent with postmenopausal status (Figure 1, Figure 2 and Figure 3). The adnexa appeared unremarkable and consistent with the patient’s age. Preoperative laboratory findings are summarized in Table 1. Tumor marker (CA 125 28 U/mL) and coagulation parameters, as well as serum estradiol and progesterone levels (estradiol 18 pg/mL and progesterone 0.7 ng/mL), were all within postmenopausal reference ranges. Given the persistence of postmenopausal bleeding and the presence of an enlarging uterine mass, exploratory curettage was performed. Histopathological analysis demonstrated an inactive, atrophic endometrium without evidence of malignancy. Following appropriate preoperative preparation and internal medicine evaluation, the patient underwent a total abdominal hysterectomy with bilateral adnexectomy in January 2025. Intraoperatively, an enlarged uterus containing a well-circumscribed nodular lesion located in the fundal region and measuring approximately 5–7 cm in diameter was observed. The adnexa appeared macroscopically normal. The operative and immediate postoperative course proceeded without complications. Microscopic examination revealed a smooth muscle tumor composed of intersecting fascicles of spindle cells with moderate cytological atypia, focal coagulative tumor necrosis, and mitotic activity of up to five mitoses per ten high-power fields. In the absence of unequivocal criteria for leiomyosarcoma, and based on the overall combination of morphological features, a diagnosis of STUMP was established (Figure 4, Figure 5, Figure 6 and Figure 7). Postoperative staging with multislice computed tomography (MSCT) of the abdomen and pelvis demonstrated no evidence of residual or metastatic disease. The patient was referred to a tertiary referral center for further management and long-term surveillance. The timeline of diagnostic procedures, surgical treatment, and follow-up visits is presented in Figure 8. At the time of writing, the patient has been followed for 13 months after surgery (January 2025), with the most recent follow-up examination performed in February 2026. During this period, she underwent three follow-up evaluations at a tertiary referral center. The follow-up protocol included clinical examination and pelvic US every six months. The most recent pelvic US (abdominal and transvaginal), performed in February 2026, showed no evidence of local recurrence. In addition, thoracic imaging was performed to exclude distant metastases, particularly pulmonary involvement; a chest CT scan conducted in January 2026 revealed no pathological findings. To date (March 2026), no clinical, radiological, or laboratory evidence of recurrence has been observed. Long-term follow-up is ongoing, with planned evaluations at 6–12-month intervals, including clinical and radiological assessment, given the potential for late recurrence in STUMP tumors.

## 3. Discussion

### 3.1. STUMP as a Diagnostic Gray Zone

The contemporary diagnostic approach, originally based on the criteria proposed by Kempson, relies on the assessment of three key histological parameters: the degree of cytological atypia, mitotic activity, and the presence of coagulative tumor necrosis [7]. Combinations of these criteria form the basis for distinguishing benign LMs from LMS. Importantly, STUMP is not defined by the presence of these criteria alone, but rather by their ambiguous, borderline, or discordant combination. In numerous cases, the findings demonstrate intermediate features that do not allow for definitive classification. Tumors with such characteristics are categorized as STUMP, highlighting the limitations of existing morphological criteria in predicting their biological behavior [8,9,10]. The definition of STUMP is based on the assessment of three key histological parameters: cytological atypia, mitotic activity, and the presence of coagulative tumor necrosis. However, variability in the interpretation of these criteria exists across the literature. Classical definitions describe STUMP as tumors exhibiting coagulative tumor cell necrosis, a mitotic count ≤ 10 per 10 high-power fields (HPFs), and none-to-mild atypia [8,9,10]. In Kempson’s scheme, tumors with higher mitotic activity (>15/10 HPF) may also be classified as STUMP [7]. Furthermore, Guntupalli et al. have distinguished mitotically active LM (defined as >5 and <19 mitoses per 10 HPF) as a benign entity separate from STUMP [11]. According to the WHO, STUMP includes uterine smooth muscle tumors with features suggesting malignant potential that do not fulfill criteria for LM or LMS [1,10]. Although most STUMP tumors exhibit a relatively indolent clinical course, their behavior remains unpredictable. The literature describes cases of local recurrence, peritoneal dissemination, and distant metastases, including involvement of the lungs and abdomen. A particular diagnostic and prognostic challenge lies in the fact that these unfavorable outcomes may occur several years after initial treatment, even in tumors that histologically demonstrate minimal atypia and low mitotic activity. This further supports the concept that STUMP is not a single entity but rather a spectrum of tumors with varying malignant potential that require an individualized clinical approach [11,12,13]. The diagnostic complexity of STUMP tumors is also reflected in significant inter- and intra-observer variability among pathologists. Differences in the interpretation of morphological criteria, particularly in the assessment of coagulative tumor necrosis and the degree of cytological atypia, may lead to variations in the classification of the same tumor as LM, STUMP, or LMS. This underscores the need for standardization of diagnostic criteria and the integration of additional prognostic parameters, including immunohistochemical and molecular markers, into the contemporary diagnostic algorithm [4,14,15]. Preoperative differentiation among LMs, STUMP, and LMS remains limited, further confirming the central role of definitive histopathological evaluation in establishing the diagnosis and planning subsequent clinical follow-up [16,17,18].

### 3.2. Differential Diagnosis in Relation to Leiomyosarcoma

The key challenge in uterine smooth muscle tumors lies in the fact that, in clinical practice, the boundary between benign LM and LMS is not established on the basis of a single “decisive” finding, but rather on a combination of morphological criteria, with recognition of situations in which these criteria are only partially present or mutually discordant. It is precisely within this zone of overlap that the diagnostic space for STUMP emerges: lesions that do not reliably meet the criteria for LMS but exhibit one or more worrisome features exceeding those of a typical LM [17,19,20]. In routine histopathological practice, differentiation between STUMP and LMS is based on the evaluation of three parameters, namely A. cytological atypia, B. mitotic index, and C. coagulative tumor necrosis, with additional assessment of growth pattern (e.g., infiltrative borders). According to the Stanford criteria, LMS typically demonstrates marked (severe) atypia, high mitotic activity (often ≥10 mitoses/10 HPF), and/or the presence of coagulative tumor necrosis, frequently accompanied by an infiltrative growth pattern [7,19]. In contrast, STUMP includes cases in which one criterion is borderline or present in isolation or where the combination of findings is insufficient for confident classification as LMS but not entirely compatible with an LM [17,19,20]. An important practical step is the distinction between “true” coagulative tumor necrosis and secondary or degenerative changes in LMs (hyalinization, infarct-type necrosis, and hemorrhagic and apoplectic changes), as misinterpretation of necrosis may artificially shift the diagnosis toward suspicion of LMS. Similarly, mitotic activity must be assessed in appropriately selected, most active areas and interpreted in correlation with atypia and necrosis, since benign or borderline entities (e.g., mitotically active LM, LM with bizarre nuclei/symplastic LM, apoplectic LM) may exhibit increased proliferative activity or focal atypia without the biological behavior of LMS. These “mimic” patterns represent one of the most common pitfalls in differential diagnosis [8,15,19]. Immunohistochemistry (IHC) may serve as a useful adjunctive tool but does not represent a standalone gold standard for distinguishing STUMP from LMS. LMS more frequently demonstrates diffuse p16 overexpression and an aberrant p53 pattern (mutant phenotype), along with a significantly higher Ki-67 index (often markedly elevated), whereas LMs typically show a wild-type p53 pattern and a very low Ki-67 index, with preserved expression of smooth muscle markers and often ER/PR positivity. STUMP most commonly retains an LM-like immunoprofile but may exhibit intermediate patterns (e.g., focal or patchy p16 expression and moderately increased Ki-67). Overlap exists: p16 and p53 positivity may also be observed in certain benign subtypes (bizarre nuclei/symplastic or apoplectic variants; mitotically active LM), which limits the specificity of IHC findings if not interpreted strictly within the morphological context [14,15,19]. The safest and most reproducible approach to differential diagnosis includes (A) primary reliance on morphological criteria (atypia–mitoses–necrosis plus growth pattern); (B) targeted use of IHC as supportive evidence when morphology is inconclusive (particularly p16/p53/Ki-67 alongside standard smooth muscle markers and hormone receptors); and (C) explicit documentation of the specific factors preventing a definitive diagnosis of LMS (e.g., necrosis without accompanying high mitotic activity and without severe atypia or 5–9 mitoses/10 HPF without additional malignant criteria) in order to ensure logical and transparent clinical follow-up and potential re-evaluation [13,17,19]. Differential diagnostic criteria for leiomyoma, STUMP, and leiomyosarcoma are presented in Table 2.

**Table 2:** Differential diagnostic criteria: leiomyoma vs. STUMP vs. leiomyosarcoma.

Characteristic	Leiomyoma (LM)	STUMP	Leiomyosarcoma (LMS)
Cytological atypia	Minimal or absent	Moderate or focal, without diffuse severe atypia	Diffuse moderate–severe atypia
Mitotic index	<5/10 HPF	Most commonly <10/10 HPF (intermediate or discordant)	≥10/10 HPF
Coagulative tumor necrosis	Absent	Focal or equivocal, without full LMS triad	Typically present
Growth pattern	Expansile, well-circumscribed	Most often well-circumscribed	Frequently infiltrative
Combination of criteria	Benign morphology	Discordant or borderline findings	≥2 major LMS criteria
IHC: p16	Negative/focal	Variable, patchy	Diffusely positive
IHC: p53	Wild-type	Most commonly wild-type	Aberrant/mutant
Ki-67	Low	Intermediate	High
Biological behavior	Benign	Unpredictable	Malignant
Risk of recurrence	Minimal	Low but real	High
Metastases	No	Rare, reported	Frequent
Definitive diagnosis	Histologically clear	Often postoperative	Histologically clear

The table summarizes key histopathological parameters, including cytological atypia, mitotic index per 10 high-power fields (HPFs), presence of coagulative tumor necrosis, growth pattern, immunohistochemical (IHC) profile (p16, p53, and Ki-67), biological behavior, risk of recurrence, metastatic potential, and criteria required for definitive diagnosis. Diagnostic categorization is primarily based on the combination of cytological atypia, mitotic activity, and coagulative tumor necrosis (the “LMS triad”) [5,9].

### 3.3. Postmenopausal STUMP—Clinical Importance and Specific Considerations

The occurrence of uterine smooth muscle tumors in the postmenopausal period requires a heightened level of clinical vigilance, as any newly detected or symptomatic uterine mass in this population is initially regarded with increased suspicion for malignancy. Unlike the reproductive period, when LMs are common and expected, postmenopausal bleeding or growth of a uterine mass represents a “red flag” that often leads to a more radical diagnostic and therapeutic approach in clinical practice. In this context, STUMP in postmenopause represents a particularly challenging entity, as the clinical presentation and patient demographic profile may mimic other uterine tumors [11,13].

The clinical presentation of STUMP in postmenopausal women most commonly includes abnormal uterine bleeding, uterine enlargement, or detection of an intrauterine mass on ultrasound examination. However, none of these symptoms is specific, and they overlap entirely with the presentation of LMS as well as with benign degenerative changes in LMs. This clinical nonspecificity further complicates preoperative assessment and often results in a decision for surgical management without a definitive preoperative histological diagnosis in the majority of cases [10,16]. In view of the above, STUMP in the postmenopausal population represents an entity of particular clinical significance. This combination of diagnostic uncertainty and potentially unpredictable biological behavior renders postmenopausal STUMP tumors especially relevant for contemporary clinical practice and underscores the need for standardized follow-up algorithms [12,17].

### 3.4. Diagnostic Pitfalls in Postmenopausal Uterine Masses

Postmenopausal uterine masses present a distinct diagnostic challenge, primarily because clinical, imaging, and limited histological assessment often fail to reliably reflect the underlying biological potential of the lesion. In this setting, both overestimation and underestimation of malignant risk may occur. One of the most relevant pitfalls is the assumption that a long-standing, previously diagnosed LM remains biologically stable after menopause. Although most LMs regress in the absence of hormonal stimulation, interval growth in postmenopausal women should not be automatically attributed to benign degenerative changes. Reported cases of STUMP and LMS initially interpreted as LM emphasize that tumor enlargement after menopause warrants careful reassessment rather than reassurance [17,20]. Imaging findings may further contribute to diagnostic uncertainty. US and even MRI can demonstrate heterogeneous echotexture, cystic or necrotic areas, and variable vascularization in both degenerating LMs and tumors with malignant potential. Currently, no imaging modality has demonstrated sufficient specificity to reliably distinguish LM from STUMP or LMS in routine clinical practice. MRI has limited value in this context and does not allow reliable differentiation of STUMP but may assist in distinguishing LM from LMS based on signal intensity characteristics [12,18]. In the present case, although histopathological examination revealed focal coagulative tumor necrosis, this feature was not identified on preoperative US. This discrepancy may be explained by the limited sensitivity of US in detecting focal or microscopic necrotic changes, particularly in heterogeneous tumors. In addition, degenerative changes in LMs may produce similar imaging appearances, further complicating the correlation between imaging and histopathological findings. Recent efforts to develop structured US-based algorithms have improved risk stratification. Overlap between benign and borderline or malignant lesions persists, limiting definitive preoperative classification [21,22]. As a result, radiological interpretation must always be correlated with clinical evolution, particularly with symptom persistence or progression. Endometrial sampling represents another frequent source of false reassurance. In patients presenting with postmenopausal bleeding, curettage primarily evaluates the endometrium and may exclude endometrial carcinoma, but it does not provide adequate information about the mesenchymal tumor component located within the myometrium [19]. Consequently, a benign or atrophic endometrial finding does not exclude the presence of a smooth muscle tumor with borderline or malignant potential, as illustrated in the present case. A further difficulty lies in the interpretation of intraoperative or limited pathological sampling. Small biopsy specimens or fragmented tissue may not capture the most diagnostically relevant areas of atypia, mitotic activity, or coagulative tumor necrosis. Definitive classification frequently requires complete tumor excision and thorough histopathological evaluation of the entire lesion [8,19,20]. Taken together, these pitfalls explain why STUMP remains predominantly a postoperative diagnosis. In postmenopausal patients, tumor growth, persistent bleeding, or discordance between clinical evolution and reassuring initial findings should prompt consideration of definitive surgical management rather than prolonged conservative observation.

### 3.5. Treatment—Surgical Approach, Extent of Surgery, and the Role of Adjuvant Therapy

The cornerstone of treatment for STUMP tumors remains surgical management. Current literature describes hysterectomy or tumor excision (myomectomy) as the principal therapeutic options, with the extent of surgery individualized according to patient age, symptomatology, tumor characteristics, and reproductive plans. Owing to the biological heterogeneity of STUMP and the variability in reported recurrence rates, a universally accepted standard of care has not been established. Therapeutic decisions are therefore typically made within a multidisciplinary framework, balancing oncologic safety and patient-specific considerations [9,14,21]. Although not applicable in the present case, fertility-sparing approaches such as myomectomy have been reported in selected patients desiring future reproduction. Available series evaluating reproductive and oncologic outcomes suggest that pregnancy after conservative management is possible, although long-term oncologic safety remains the primary concern [23]. However, this approach requires thorough preoperative counseling and strict postoperative surveillance. The rationale lies in the possibility of residual disease, local recurrence, or delayed relapse, which in rare cases may demonstrate more aggressive biological behavior. Published series emphasize that fertility-sparing management should be accompanied by careful follow-up and a low threshold for definitive surgery in the presence of suspicious clinical or radiological findings [10,11]. A recent multicenter analysis comparing fertility-sparing surgery with hysterectomy reported comparable overall survival but highlighted the necessity of rigorous postoperative monitoring in conservatively treated patients [24]. In postmenopausal patients and in women without reproductive plans, a more radical surgical strategy is generally favored. Total hysterectomy, frequently combined with bilateral adnexectomy, is considered a reasonable and oncologically prudent approach, particularly when persistent bleeding, interval tumor growth, or atypical imaging features raise concern for malignancy. This strategy allows for complete histopathological evaluation of the entire lesion and minimizes the likelihood of residual disease. In the present case, total abdominal hysterectomy with bilateral adnexectomy represented a rational and literature-supported management choice in a postmenopausal patient presenting with persistent bleeding and an enlarging uterine mass [12,13,17]. An additional surgical consideration concerns the method of specimen extraction. When STUMP or LMS cannot be confidently excluded preoperatively, tumor fragmentation, particularly power morcellation, has been associated with concern regarding potential intraperitoneal dissemination and altered recurrence patterns. Although high-quality prospective data are lacking and the precise impact on long-term outcomes in STUMP remains difficult to quantify, retrospective analyses and systematic reviews support a cautious approach in cases with atypical features [4,8,19]. Broader uterine sarcoma literature similarly emphasizes the potential oncologic implications of morcellation when malignancy cannot be excluded preoperatively, supporting a cautious specimen-extraction strategy [25]. En bloc removal of the specimen, whenever technically feasible, enables a comprehensive histopathological assessment and theoretically prevents the spread of tumor tissue. Careful surgical planning and individualized risk assessment are therefore essential, especially in postmenopausal women or in tumors demonstrating interval growth. With regard to adjuvant therapy, currently available evidence does not support routine chemotherapy or radiotherapy following primary surgical treatment of STUMP. Due to the rarity of the entity and the absence of randomized trials, recommendations are largely derived from retrospective data and extrapolation from LMS management. Adjuvant treatment is generally reserved for cases of recurrence, metastatic disease, or histologically high-risk patterns, and decisions are made on an individual basis in specialized centers [13,14,17].

### 3.6. Recurrence and Metastatic Potential

The biological behavior of STUMP tumors is characterized by marked variability, which represents the main reason for their designation as a distinct diagnostic category among uterine smooth muscle tumors. Although the majority of cases in clinical practice follow an indolent course, available series indicate that recurrence occurs in approximately 7–27% of patients, while some authors report rates of up to 30–36%, depending on the duration of follow-up and the diagnostic criteria applied. A recent long-term evaluation of surgical outcomes further confirmed that recurrence may occur despite apparently adequate primary treatment and emphasized the importance of extended surveillance beyond the initial postoperative years [26]. These data confirm that the risk of recurrence is not negligible and may persist even after initially radical surgical treatment, thereby justifying the need for long-term and structured postoperative surveillance [11,13,21]. The interval to recurrence is highly heterogeneous. In most published series, the median time to recurrence ranges between 40 and 60 months. Early recurrences within the first two to three years, as well as late recurrences occurring after five or even more than ten years following the initial surgery, have been described. This temporal pattern distinguishes STUMP from a typical LM and further underscores the need for prolonged clinical and radiological follow-up, regardless of initially favorable histological characteristics [12,19]. In a smaller proportion of cases, recurrence may follow a more aggressive course, including the development of distant metastases or histological transformation into LMS. The most frequently reported sites of metastatic disease are the lungs and intra-abdominal structures, while the overall incidence of metastases in most series is considered low and generally below 10%. The occurrence of metastatic disease even in tumors that initially did not meet all criteria for malignancy further highlights the heterogeneity of this group and the limitations of morphological assessment in predicting clinical behavior [11,21]. Multicenter data analyzing cases initially classified as LM but later associated with recurrence or metastasis further illustrate the diagnostic and prognostic complexity of STUMPs [27]. Attempts to identify reliable prognostic factors have not yet resulted in a universally accepted risk stratification model. Some studies have suggested that specific histological features may be associated with recurrence risk. In particular, tumors exhibiting significant cytological atypia alone or coagulative tumor cell necrosis alone have been associated with an increased risk of recurrence, whereas tumors characterized solely by a high mitotic index (>10 mitoses/10 HPF) have not consistently demonstrated recurrence [4,11,13]. Detailed clinicopathological analyses have proposed additional histological parameters potentially associated with adverse outcomes. Reproducibility and predictive consistency across cohorts remain limited [28]. The presence of tumor necrosis, a higher mitotic index, and more pronounced cytological atypia have been associated with an increased likelihood of recurrence in certain studies; however, available data remain inconsistent and do not allow for precise individual prognostication. Research emphasizes that all patients with a diagnosis of STUMP require long-term follow-up, irrespective of the initial histological findings or the extent of primary surgical treatment [10,11,13]. Given this pattern of behavior, assessment of the risk of recurrence and metastatic spread represents a central element of postoperative management in patients with STUMP tumors. The potential for late recurrence, the absence of reliable prognostic parameters, and the variability of clinical course distinguish this group of tumors from classical benign LMs and justify the need for long-term surveillance in specialized centers [12,13]. Recent institutional series with long-term follow-up further support that recurrence may occur after prolonged disease-free intervals, reinforcing the need for extended surveillance [29]. Although no universally accepted surveillance protocol currently exists for patients diagnosed with STUMP, available data on recurrence rates and timing of relapse allow for the formulation of a pragmatic follow-up strategy. Based on published recurrence patterns and expert recommendations, a structured surveillance approach may be considered, particularly in patients with borderline histological features or fertility-sparing surgery (Table 3).

### 3.7. Immunohistochemical and Molecular Markers

Despite advances in the histopathological classification of STUMP, morphological criteria remain the cornerstone of the diagnostic process, while immunohistochemical and molecular markers currently play a primarily adjunctive and prognostic role. Numerous studies indicate that no single immunohistochemical marker can reliably distinguish STUMP from LMS or atypical LMs. Combinations of selected markers may contribute to a more precise assessment of the proliferative and biological potential of these tumors [14,15,18]. Molecularly informed immunohistochemical algorithms have been proposed to improve diagnostic precision. These approaches have not yet replaced morphology as the decisive diagnostic standard [30]. The most frequently investigated markers include p16, p53, and Ki-67, which are cited in the literature as potential indicators of more aggressive biological behavior. LMS typically demonstrate diffuse and strong p16 expression, an aberrant p53 pattern, and a high Ki-67 proliferative index, whereas benign LMs exhibit low Ki-67 levels and a preserved wild-type p53 pattern. STUMP tumors most often fall between these two extremes, with variable and frequently heterogeneous expression profiles, reflecting their intermediate biological nature [15,19]. Some studies suggest that increased Ki-67 proliferative activity and aberrant expression of p16 or p53 may be associated with a higher risk of recurrence; however, the available data remain inconsistent and do not allow for reliable individual prognostic stratification. A systematic review and meta-analysis assessing p53, p16, and Ki-67 in STUMP supports their potential prognostic value while also highlighting variability across studies and the continued need for morphology-driven interpretation [31]. For this reason, immunohistochemical findings must be interpreted in relation to morphological criteria rather than as independent diagnostic or prognostic parameters [13,18]. In addition to conventional immunohistochemical markers, contemporary research has focused on the molecular characterization of uterine smooth muscle tumors. Various genetic and epigenetic alterations have been described, including aberrations in genes involved in cell cycle regulation and proliferation. To date, no molecular marker has entered routine clinical practice as a reliable prognostic or diagnostic tool for STUMP. Recent studies have further explored the molecular landscape of uterine smooth muscle tumors, identifying recurrent alterations in LM driver genes such as MED12, HMGA2, and FH. In addition, loss of ATRX and DAXX expression, as well as increased transcriptomic complexity, have been associated with more aggressive tumor behavior and may contribute to improved risk stratification in STUMP. These markers are not yet part of routine clinical practice and require further validation [20]. The absence of standardized molecular criteria further confirms that STUMP remains primarily a morphological and clinical diagnostic category [14,19]. Accordingly, the current approach involves the integration of morphological assessment, immunohistochemical profile, and clinical context, with a strong emphasis on long-term patient follow-up regardless of the initial immunohistochemical findings. Although IHC and molecular markers hold potential to improve prognostic stratification in the future, their role at present remains supportive and complementary within comprehensive histopathological evaluation [13,15,18]. In the present case, extended immunohistochemical profiling was not performed, as the morphological features were considered sufficient to establish the diagnosis. Nevertheless, current evidence suggests that markers such as p16, p53, and Ki-67 may provide additional contextual information in diagnostically ambiguous tumors, particularly when histological findings are borderline. Importantly, these markers cannot replace careful evaluation of cytological atypia, mitotic activity, and coagulative tumor necrosis, which remain the decisive criteria for classification [14,15,19].

### 3.8. Clinical Implications and the Role of the Presented Case

The presented case clearly illustrates the fundamental diagnostic challenge of uterine smooth muscle tumors: the limited ability to reliably differentiate preoperatively between LMs, STUMP tumors, and LMS. Despite the typical ultrasound appearance of a myomatous lesion and a normal endometrial finding following fractional curettage, the final diagnosis of a smooth muscle tumor of uncertain malignant potential was established only after definitive histopathological analysis of the surgical specimen. This finding is expected, given that curettage most often yields only endometrial tissue, while the mesenchymal tumor component remains inaccessible for adequate evaluation. This confirms that clinical, radiological, and partial histopathological assessments are frequently insufficient for reliable evaluation of malignant potential and that STUMP, in the majority of cases, remains a postoperative diagnosis [17,19]. The particular significance of this case arises from the patient’s postmenopausal status and clinical presentation with persistent uterine bleeding. In this population, any newly detected uterine mass requires heightened oncologic vigilance due to the greater likelihood of malignancy, which in clinical practice often leads to a more radical surgical approach. At the same time, the absence of reliable preoperative criteria for identifying STUMP tumors means that assessment of malignant potential and definitive classification can be made only after complete histopathological evaluation, further underscoring the importance of thorough pathological analysis and multidisciplinary decision-making in treatment planning [12,13]. The morphological characteristics of the tumor in this case (moderate cytological atypia, the presence of focal necrosis, and mitotic activity at the borderline of malignancy) are typical of the intermediate spectrum of uterine smooth muscle tumors and confirm the diagnostic complexity of the STUMP category. At the same time, the absence of reliable prognostic parameters enabling precise assessment of recurrence risk highlights the need for long-term and structured follow-up, regardless of the initially performed radical surgical treatment [11,21]. In the postmenopausal population, total hysterectomy with bilateral adnexectomy represents a rational and oncologically safe therapeutic option in patients presenting with symptomatic bleeding and a suspected uterine tumor mass. This approach allows for complete histopathological evaluation, reduces the risk of residual disease, and provides an adequate basis for subsequent oncologic surveillance. The presented case confirms that the combination of nonspecific clinical presentation, limitations of preoperative diagnostics, and potentially unpredictable biological behavior renders STUMP a clinically significant entity in contemporary practice, particularly in the postmenopausal population [13,14]. Overall, this case emphasizes the need for caution in the interpretation of seemingly benign uterine tumors in the postmenopausal period, as well as the importance of an individualized therapeutic and surveillance strategy. Recognition of the limitations of preoperative diagnostics, adequate histopathological evaluation, and inclusion of the patient in a long-term follow-up program at a tertiary center represent key elements of modern management of STUMP tumors [12,17]. In the present case, mild laboratory abnormalities, including a slightly elevated monocyte count and reduced total protein levels, were observed preoperatively and after STUMP was diagnosed. These findings are nonspecific and may be associated with a variety of benign or systemic conditions, such as low-grade inflammation or nutritional status. To our knowledge, there is currently no evidence supporting a diagnostic or prognostic role of these laboratory parameters in STUMP, and they were therefore considered incidental findings.

## 4. Conclusions

STUMP remains one of the most complex and diagnostically challenging entities in contemporary gynecologic pathology. The difficulty lies not in its rarity but in the persistent uncertainty regarding its biological behavior and the limitations of current tools in predicting clinical outcomes. Preoperative differentiation from benign LM or LMs remains unreliable, as clinical presentation, imaging findings, and endometrial sampling cannot adequately assess the mesenchymal component of the tumor. In most patients, a definitive diagnosis is established only after complete surgical excision and comprehensive histopathological evaluation. The present case underscores a clinically significant scenario: interval enlargement of a previously presumed LM accompanied by persistent postmenopausal bleeding. In a hormonally quiescent patient, such findings should not be considered reassuring. Postmenopausal growth of a uterine mass represents a biologically relevant signal that warrants careful reassessment and, when appropriate, definitive surgical management. Even in the absence of overtly malignant imaging characteristics, oncologic vigilance remains justified. Although many STUMP cases demonstrate a relatively indolent course, available evidence confirms a non-negligible risk of late recurrence and, in rare cases, distant metastatic spread. Importantly, recurrence may occur several years after initial treatment and cannot be reliably predicted solely on the basis of morphological features. For this reason, the absence of universally accepted surveillance protocols represents a clinically meaningful gap in gynecologic oncology practice. Structured, long-term follow-up strategies are essential until validated prognostic markers and standardized management pathways become available. Ultimately, optimal management of STUMP requires integration of clinical judgment, careful surgical planning, detailed pathological assessment, and individualized risk evaluation. Recognition of postmenopausal tumor growth as a potential indicator of underlying biological uncertainty may facilitate timely intervention, reduce the risk of delayed diagnosis, and contribute to improved long-term patient monitoring. Continued efforts toward standardized surveillance algorithms and improved diagnostic stratification remain necessary to address the unresolved challenges associated with this intermediate tumor category.

## Figures and Tables

**Table 1:** Preoperative laboratory findings.

Parameter	Result	Reference Range	Unit
Leukocytes	8.6	4.0–10.0	×10^9^/L
Neutrophils	63.9	50.0–70.0	%
Lymphocytes	25.5	25.0–40.0	%
Monocytes	7.4	2.0–6.0	%
Eosinophils	2.6	2.0–4.0	%
Basophils	0.6	<1.0	%
Erythrocytes	4.42	3.80–5.80	×10^12^/L
Hemoglobin	142	115–160	g/L
Hematocrit	0.413	0.370–0.470	L/L
MCV	93.5	80–100	fL
MCH	32.2	27–32	Pg
MCHC	344	320–360	g/L
Platelets	276	150–500	×10^9^/L
CRP	3.12	<5.0	mg/L
AST	17	<31	U/L
ALT	16	<34	U/L
LDH	190	<247	U/L
Total bilirubin	7.4	2–21	µmol/L
Creatinine	53	58–96	µmol/L
Urea	4.5	2.8–7.2	mmol/L
Total proteins	59.6	66–83	g/L
Albumin	38.3	34–53	g/L

**Table 3:** Proposed follow-up algorithm for patients with uterine STUMP after primary surgical treatment.

Time from Surgery	Clinical Examination	Pelvic Imaging (US/MRI)	Thoracic Imaging	Additional Considerations
0–24 months	Every 6 months	Every 6 months	Annually	Higher vigilance in cases with necrosis, higher mitotic index, or fertility-sparing surgery
25–60 months	Annually	Annually	Annually	Consider MRI if ultrasound findings are equivocal
>5 years	Individualized	Individualized	If clinically indicated	Long-term follow-up advised due to reported late recurrences
At any time if symptoms occur	Immediate evaluation	Targeted imaging	Targeted imaging	Assess for local recurrence or distant metastasis (lungs most common site)

Legend: The proposed follow-up schedule is based on published recurrence patterns and reported timing of local and distant relapse in STUMP cases. Surveillance intensity should be individualized according to histopathological features and surgical approach [4,8,11,19].

## Data Availability

Data presented in this study are available from the corresponding author upon request.

## Abbreviations

The following abbreviations are used in this manuscript:

LM	Leiomyoma
LMS	Leiomyosarcoma
STUMP	Smooth muscle tumor of uncertain malignant potential
HPF	High-power field
IHC	Immunohistochemistry
ER	Estrogen receptor
PR	Progesterone receptor
KI-67	Proliferation index
MSCT	Multislice computed tomography
H&E	Hematoxylin and eosin
